# Al_2_O_3_-Cu Substrate with Co-Continuous Phases Made by Powder Sintering Process

**DOI:** 10.3390/ma11081477

**Published:** 2018-08-20

**Authors:** Shuangxi Wang, Haifeng Lan, Wenjun Wang, Gaoshan Liu, Dan Zhang

**Affiliations:** College of Engineering, Shantou University, Shantou 515063, China; 16hflan@stu.edu.cn (H.L.); 14wjwang@alumni.stu.edu.cn (W.W.); 12gsliu@alumni.stu.edu.cn (G.L.); 13dzhang@alumni.stu.edu.cn (D.Z.)

**Keywords:** thermal conductivity, ceramic-based substrate, co-continuous metal phase, Cu fiber, powder sintering process

## Abstract

Ceramic-Al substrates with co-continuous ceramic and metal phases, which exhibit high thermal conductivity and compatible coefficient of thermal expansion (CTE), have been widely investigated through the process of die-casting. In this research, a kind of powder sintering process was proposed for fabricating ceramic-Cu composite substrates with co-continuous phases. Copper fiber (Cu_f_) has excellent thermal conductivity and large aspect ratio, making it an ideal material to form bridging network structures in the ceramic-Cu composite. To maintain the large aspect ratio of Cu_f_, and densify the composite substrate, ZnO-SiO_2_-CaO glass was introduced as a sintering additive. Both Al_2_O_3_/glass/Cu_f_ and Al_2_O_3_/30glass/Cu_p_ composite substrates were hot-pressed at 850 °C under 25 MPa. Experimental results showed that the thermal conductivity of Al_2_O_3_/30glass/30Cu_f_ composite substrate was as high as 38.9 W/mK, which was about 6 times that of Al_2_O_3_/30glass; in contrast, the thermal conductivity of Al_2_O_3_/30glass/30Cu_p_ composite substrate was only 25.9 W/mK. Microstructure observation showed that, influenced by hot press and corrosion of molten ZnO-SiO_2_-CaO glass, the copper fibers were deformed under hot-pressing, and some local melting-like phenomena occurred on the surface of copper fiber at 850 °C under 25 MPa. The molten phase originating from surface of Cu_f_ welded the overlapping node of copper fibers during cooling process. Finally, the interconnecting metal bridging in ceramic matrix was formed and behaved as a rapid heat-dissipating channel, which is similar to substrates prepared through die-casting process by porous ceramic and melted Al.

## 1. Introduction

As the fourth generation of lighting sources, light-emitting diodes (LEDs) have been widely applied in fields of automobiles, urban construction, screen displays, and traffic lights, owing to their advantages of long lifetime, high response speed, small size, low power consumption, and environmental friendliness [1]. However, although LED is advertised as a cold lighting source, the photoelectric conversion efficiency of LED lamp is only about 20% to 30% [2]. Most electrical energy is still converted to heat. If heat is not managed appropriately, it always results in temperature increase in the chip and the stability, efficiency and lifetime of the LEDs will be reduced [3]. Therefore, thermal dissipation is a very important property of LEDs. The packaging substrate is the main path for heat dissipation in high-power LEDs [4]. The higher the thermal conductivity of the packaging substrate, the better is the LED quality [5].

LED packaging substrates should exhibit high thermostability, high thermal conductivity, high strength, and compatible coefficient of thermal expansion (CTE) with chips [6]. Thermal conduction is usually classified as phonon thermal conduction, photon thermal conduction, or electron conduction [7]. The thermal conductivity of plastic substrates is usually too low for high-power LEDs [8]. Although metal substrates have high thermal conductivity owing to free electrons, their CTE is too high to match that of silicon chips. Ceramics are covalent materials, and the electrons of covalent compounds are restrained. Thermal conduction in ceramics always occurs through lattice vibration. Comparatively, AlN ceramic substrate has advantages over plastic and metal substrates in terms of the insulation properties and CTE. Unfortunately, AlN ceramic substrate is too expensive for wide industrial application. To maintain a low expansion coefficient and reduce the cost of substrates, some ceramic and metal composite substrates have recently been investigated for application in high-power LEDs. Al and Cu are preferred as thermally enhanced materials [9,10]. M. Chmielewski et al. [11] investigated Cu-AlN metal matrix composites with a ceramic volume fraction between 0.1 and 0.4 prepared by a hot-pressing method in vacuum, the experimental results showed that 60Cu-40AlN composites exhibited a low CTE and high thermal conductivity coefficient of 10 × 10^−6^/K and 164.4 W/mK, respectively. Chen et al. [12] fabricated SiC/Al composites with thermal conductivity of around 270 W/mK by a vacuum hot pressing (VHP) process at 655 °C. Wang et al. [13] fabricated 3D-SiC/Al-Si-Mg interpenetrating composite (IPC) with three dimensional mutually interpenetrated structure by mold-forming and pressureless infiltration method. The CTE and thermal conductivity coefficient of the obtained IPC were 7.03 × 10^−6^/K (RT~300 °C) and 233.6 W/mK, respectively.

Melt infiltration is one of the preferred techniques for fabricating co-continuous ceramic-metal composites. This process requires fabricating porous ceramic preform first, after which the liquid alloy simultaneously reacts with and penetrates the ceramic preform [14,15]. However, the process for preparing porous ceramic preform is very complicated. Moreover, owing to the poor wettability and poor physical compatibility between metal and ceramic, it is difficult to control the quality of the substrate prepared by die-casting or pressureless infiltration in mass production.

In this paper, a kind of powder sintering process was proposed for fabricating ceramic-Cu composite substrates with co-continuous phases. Copper fiber (Cu_f_) was used with a hot-press process to fabricate an Al_2_O_3_/glass/Cu substrate with continuous metal. Compared with traditional die-casting or molten-metal infiltration processes, the advantages of this process lie in that the continuous metal phase is more homogeneous in the ceramic matrix, and it is much simpler and more efficient than traditional die-casting infiltration processes.

## 2. Experiment

### 2.1. Materials and Fabrication Process

In this study, alumina powders were provided by Jingrui New Materials Co., Ltd., Xuan Cheng, China (D50 = 3 µm, 99%). Copper fibers (D50 = 2 µm, aspect ratio = 10–20, 99%) and copper powders (D50 = 2.8 µm, 99%) were provided by Hongwu Nano New Materials Co., Ltd., Xuzhou, China. ZnO-SiO_2_-CaO glass additive (ZSC, D50 = 3 µm, Tg = 600 °C) containing fewer alkali metal ions, was made by ourselves to aid the densification of Al_2_O_3_ composites by low temperature sintering. All ZnO, SiO_2_, CaCO_3_, Al_2_O_3_, TiO_2_, MgO and Na_2_CO_3_ reagents used were chemically pure. The composition of ZnO-SiO_2_-CaO glass is shown in Table 1.

The raw materials were milled and mixed homogeneously. Then, the powders were dried and melted in an alumina crucible at 1230 °C for 60 min. Next, the molten glass was poured into deionized water. Finally, the glass frits were milled in Al_2_O_3_ jars with ZrO_2_ balls at the speed of 80 r/min for 40 h.

**Figure 1:**
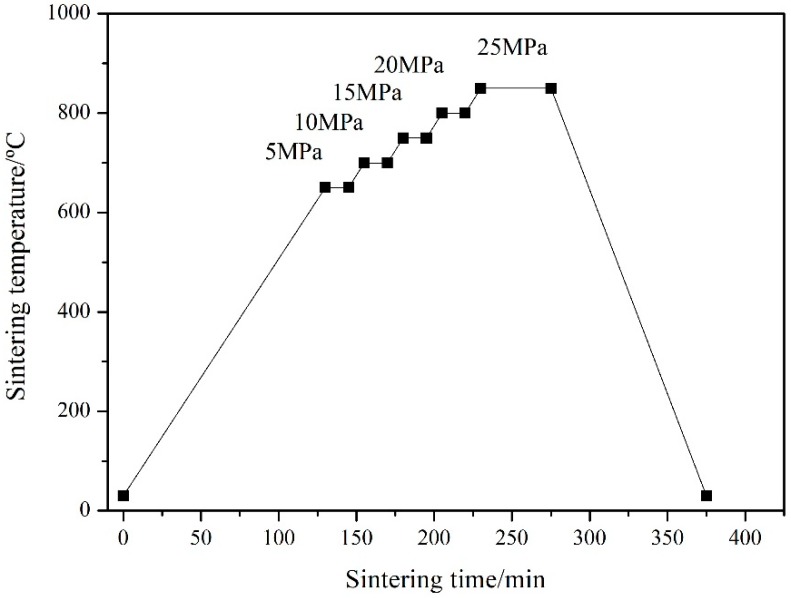
Schematic of the hot-pressing process.

Based on the volume content and shape of Cu in Al_2_O_3_/30glass/Cu composites, the experimental samples were designed as two contrasting groups, as shown in Table 2. In the traditional hot-press process, fibers or whiskers in the matrix are rotated under pressure and preferentially aligned parallel to the sample’s surface. To avoid the preferential alignment of the copper fiber and form a three-dimensional (3D) network in the Al_2_O_3_/glass green matrix [16], a hot-press process with multi-stage pressure was employed, as shown in Figure 1.

The raw materials were mixed according to the proportions listed in Table 2 and milled in ethyl alcohol for 2 h. After drying and sieving, the mixture was placed in a graphite die with inner diameter of φ30 mm and height of 100 mm, and loaded in a hot-pressed sintering furnace under N_2_ atmosphere (FVPHP-R-10 FRET-40, Fujidempa Kogyo, Osaka, Japan). About five samples with size of φ30 mm × 3 mm could be fabricated within each furnace. To form a 3D net of Cu_f_ in the composite, the pressure was applied in stages. The sintering process involved the following stages: (1) pre-sintering from room temperature to 650 °C in a stress-free manner; (2) sintering from 650 °C to 700 °C under 5 MPa (maintained for 15 min); (3) sintering from 800 °C to 850 °C under 25 MPa at a heating rate of 5 °C/min (maintained for 50 min), as shown in Figure 1. By a ceramic cutting machine (IsoMet 1000, Buehler, Lake Bluff, IL, USA), the sintered substrates with size of φ30 mm × 3 mm were cut into samples of 25 mm × 5 mm × 3 mm for CTE measuring or 10 mm × 10 mm × 1.5 mm for thermal conductivity.

### 2.2. Measurements

The relative density of the sintered specimens was measured by utilizing Archimedes’ principle. The thermal conductivity of the composites was measured using a laser thermal conductance instrument (LFA457/2/G, Netzsch, Selb, Germany). The final measurement result was the average of 5 testing values. The CTE of the composites was measured by a thermal dilatometer (DIL402C, Netzsch, Selb, Germany). Phase identification of the composites was carried out by X-ray diffraction (XRD, D/max2000v/pc, Rigaku, Toyko, Japan). The X-ray source was Ni-filtered CuKα radiation, the X-ray source operating voltage was 40 kV. The microstructure and element distribution of the sintered composites was characterized by scanning electron microscopy (SEM, ULTRA PLUS-43-13, Zeiss Ultra Plus, Jena, Germany) and transmission electron microscopy (TEM, Talos F200, FEI, Hillsboro, OR, USA).

## 3. Results and Discussion

### 3.1. Relative Density of the Composites

**Figure 2:**
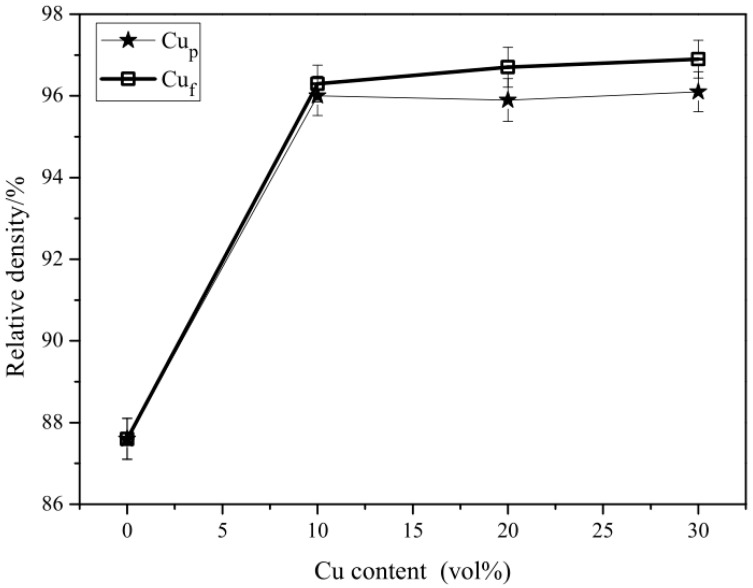
Relative density of Al_2_O_3_/30galss/Cu as a function of Cu content.

Glass additives usually behave as a liquid phase to lower the sintering temperature of Al_2_O_3_ composites. In this research, 30 vol % ZnO-SiO_2_-CaO glasses were added to the Al_2_O_3_/glass/Cu composite to ensure the sintering temperature was below 850 °C. Different contents of copper (either copper fibers material or copper particles material) from 0 to 30 vol % were added to the Al_2_O_3_/glass/Cu composites. The glass additives were expected to melt when sintered at 850 °C. As can be seen from Figure 2, the relative density of the Al_2_O_3_/30glass composite without Cu was only 87.6%. The relative density of Al_2_O_3_/30glass/Cu_f_ and Al_2_O_3_/30glass/Cu_p_ composites both increased dramatically when adding 10 vol % Cu_f_ or Cu_p_ into the Al_2_O_3_/30glass composite. This is because both copper phase and glass phase are deformable during hot-pressing at 850 °C, which is beneficial for the densification of Al_2_O_3_/glass/Cu composite. Because the volume of glass phase in these composites is constant, the greater the content of copper phase, the lower the content of Al_2_O_3_ will be. Thus, relative density is improved with the increase of Cu phase content. It is worth noting that the relative density of Al_2_O_3_/30glass/Cu_f_ is slightly higher than that of Al_2_O_3_/30glass/Cu_p_. This abnormal result is related to the presence of ZnO-SiO_2_-CaO glass phases. Generally, the ZnO-SiO_2_-CaO glass phase sintered under 850 °C behaves as if in liquid state. When the sintering temperature surpasses 600 °C (Tg of ZnO-SiO_2_-CaO glass), the glass begins to melt, and liquid glass still has high viscosity. It fills the intergranular pores of Al_2_O_3_ particles, reducing the possibility of gas escaping, and some pores are enclosed in the Al_2_O_3_/30glass composite. The plastic deformation of Cu phase could significantly increase the densification of Al_2_O_3_/glass/Cu composites under hot-press conditions. Moreover, when adding some content of Cu_f_ to the Al_2_O_3_/glass/Cu_f_ composites, the liquid glass had a similar volume and viscosity to those of the Al_2_O_3_/30glass/Cu_p_ composite. However, the liquid glass was able to infiltrate along the surface of the Cu fiber, accelerating the infiltrating speed and extending infiltrating range of the Al_2_O_3_/glass/Cu_f_ composites, and providing a spatial structure for the inner gas to escape.

### 3.2. Microstructure of the Composites

**Figure 3:**
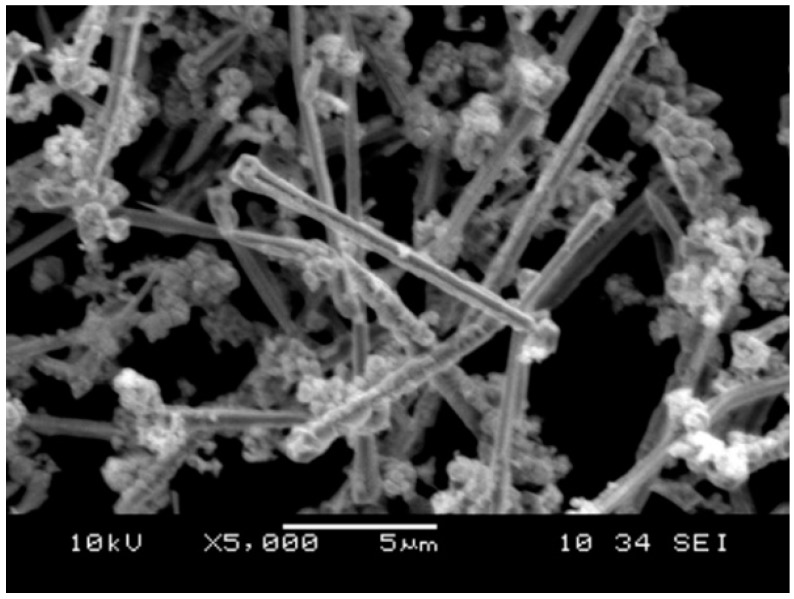
Morphology of the Cu fibers raw materials.

**Figure 4:**
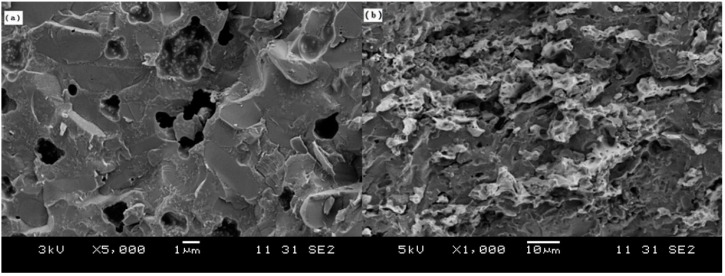
Microstructure of the fracture surface of the composite parallel to the pressure direction: (**a**) Al_2_O_3_/30glass; (**b**) Al_2_O_3_/30glass/30Cu_f_.

**Figure 5:**
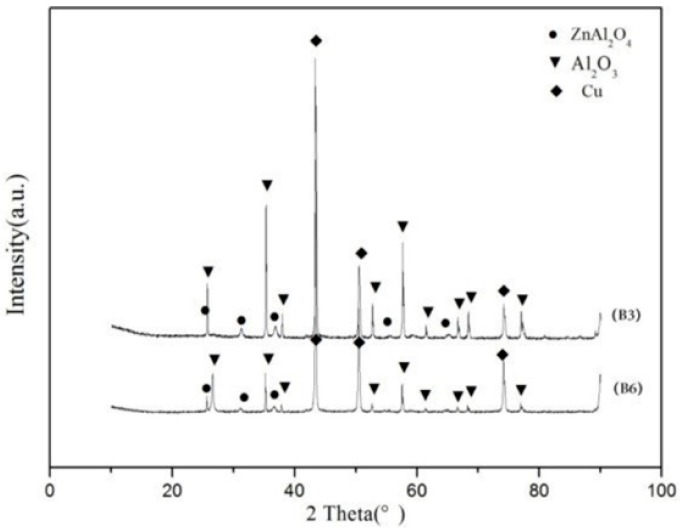
XRD spectrum of Al_2_O_3_/30glass/30Cu_f_.

Figure 3 shows the morphology of the copper fiber materials. It can be seen that the diameters of the copper fibers were about 2 µm and there were some Cu particles present in the copper fiber raw material. The melting point of Cu is 1083 °C. When the powder mixture of Al_2_O_3_/glass/Cu_f_ was hot-pressed from room temperature to 850 °C (which is far below the melting point of Cu) under a nitrogen atmosphere, the copper fibers would overlap with each other and form a 3D net. However, as shown in Figure 4, no straight fibers were observed in the fracture surface of the Al_2_O_3_/30glass/30Cu_f_ composite. It can be seen that there existed some vermiform phase in the fracture surface of the Al_2_O_3_/30glass/30Cu composite parallel to the pressure direction. The XRD result showed that there were only peaks of copper phase present in the XRD pattern (Figure 5).

**Figure 6:**
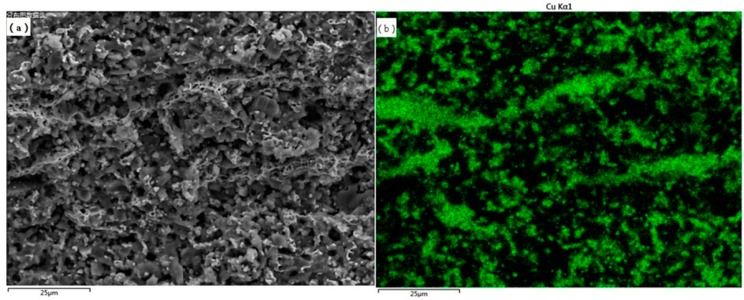
Cu distribution in the fracture surface of Al_2_O_3_/30glass/30Cu_f_ substrate parallel to the pressure direction: (**a**) Microstructure; (**b**) Distribution of Cu element marked by green.

**Figure 7:**
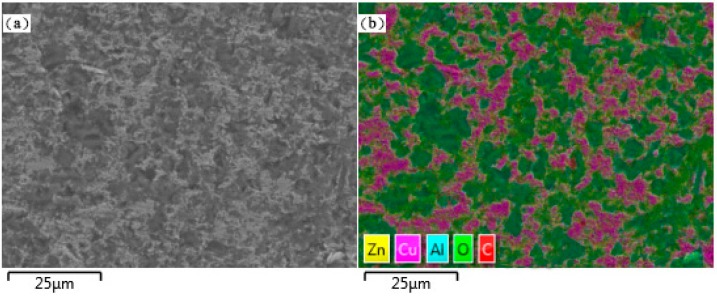
Cu distribution in the polished surface of Al_2_O_3_/30glass/30Cu_f_ substrate perpendicular to the pressure direction: (**a**) Microstructure; (**b**) Distribution of Cu element marked by magenta.

The distribution of Cu element on the fracture surface of the Al_2_O_3_/30glass/30Cu_f_ composite parallel to the pressure direction was characterized by energy dispersive X-ray spectroscopy (EDX), as shown in Figure 6b. The trace of copper became continuous in the composite, which is highly consistent with the microstructure of the Al_2_O_3_/30glass/30Cu_f_ composite (Figure 6a). Figure 7b shows the distribution of Cu element on the polished surface of the Al_2_O_3_/30glass/30Cu_f_ composite perpendicular to the pressure direction. It is worthy of note that most of the Cu element is distributed dotted on the polished surface, which means that most copper fibers are tilted at an angle with the surface of the substrate. Combined with Figure 6 and Figure 7, it could be concluded that with this process, most copper fibers successfully avoid preferential orientation under hot-pressed conditions. The 3D connected net was formed by Cu fibers’ bridging with each other.

**Figure 8:**
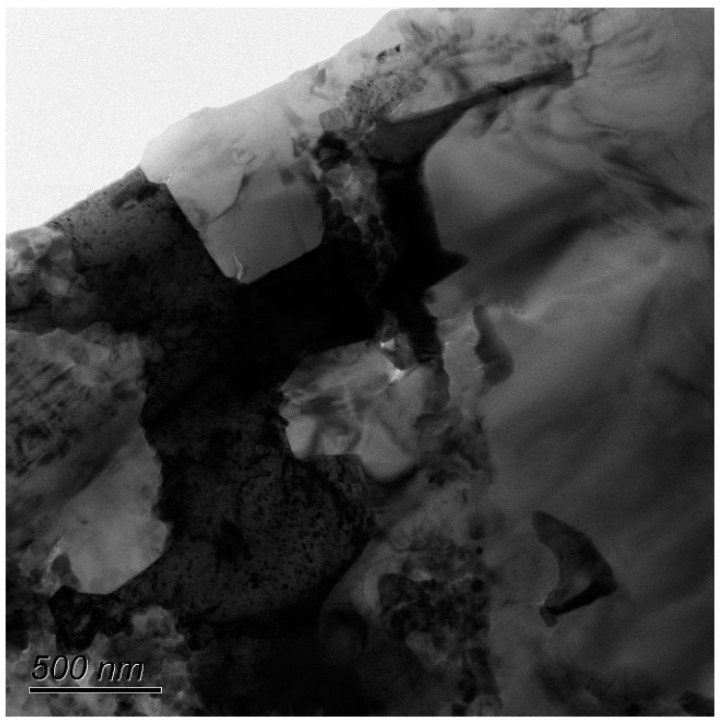
Bright field TEM micrograph of the Al_2_O_3_/30glass/30Cu_f_ composite.

**Figure 9:**
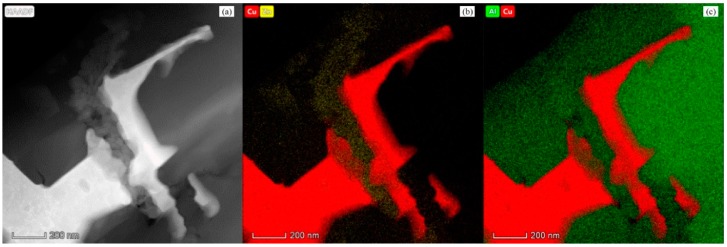
Element distributions of Al_2_O_3_/30glass/30Cu_f_ substrate: (**a**) HAADF-STEM micrograph; (**b**) Distribution of Cu and Zn elements marked by red and yellow, respectively; (**c**) Distribution of Cu and Al elements marked by red and green, respectively.

Figure 8 shows the bright field TEM micrograph of the Al_2_O_3_/30glass/30Cu_f_ composite. In this figure, it can be clearly seen that the Cu phases (dark) are deformed around the alumina (bright) and glass phases (gray). There are no pores enclosed in the interface between Cu_f_ and alumina or the interface between Cu_f_ and glass. The combination of these phases is very tight. A network structure is apparent through the overlapping of the copper fibers. Figure 9a is a local enlarged HAADF-STEM micrograph of Figure 8. Please note that compared with the interface between alumina and copper fibers, the boundary of the copper fiber and glass is much rougher. In contact with the vermiform morphology of Cu characterized by SEM in Figure 6 and Figure 7, it can be deduced that, influenced by melted glass etching and hot-press, both plastic deformation and chemical-mechanical interaction happened to the local surface of the copper fiber at 850 °C (which is far below the melting point of copper, 1083 °C). At the beginning of the hot-press, copper fibers were deformed under a pressure of 25 Mpa, and their surface oxide layers were destroyed by glass powders and Al_2_O_3_ powders. Along with the increasing of sintering temperature, the copper fibers under high outside pressure were corroded by molten glass at high temperature. Then, the melting of the local surface in the overlapping node of Cu fibers can result in a welding effect, which would significantly promote the thermal conduction ability of the Al_2_O_3_/30glass/30Cu_f_ composite. Figure 9b,c shows the distributions of Cu, Zn and Al elements in Al_2_O_3_/30glass/30Cu_f_ composite by EDX. The distribution zone of Zn corresponds to glass phase. From Figure 9a, it can be seen that there is a rough gray layer existing between the copper fiber and the glass phase.

Therefore, it could be proved that the Cu_f_ deformed and appeared as melting-like phenomena on the local surface contacting with glass when the composite was hot-pressed at 850 °C under nitrogen atmosphere. The local surface melting-like phenomenon of Cu_f_ at 850 °C under hot-pressing may be caused by the combined influences of relative high pressure and erosion of the molten glass. The surface and fracture microstructure of the Al_2_O_3_/30glass/30Cu_f_ composite shows that the vermiform Cu is interconnected and forms a continuous 3D network, which is very similar to the ideal microstructure of the co-continuous ceramic-metal composites obtained from die-casting or reactive metal penetration process [15]. A synthesis of the conclusions drawn from the melting points of glass and copper fiber, as well as the final microstructure, the sintering procedure of Al_2_O_3_/glass/Cu_f_ could be stated as follows:

(1) At low temperature, the green body of Al_2_O_3_/glass/Cu_f_ was formed, the Cu fiber deformed and the oxide layer on the surface of copper fiber was destroyed by glass and Al_2_O_3_ particles under outside pressure.
(2) ZnO-SiO_2_-CaO glass melted when the sintering temperature exceeded 600 °C.
(3) Melted glass infiltrated along the Cu fiber surface and inner gas escaped due to the spatial structure of fibers. The local surface of copper fibers corroded by the combined alternative influences of relative high pressure and erosion of the molten glass when the sintering temperature approached 850 °C.
(4) The ceramic green body gradually finished densification during the hot-pressing process.
(5) The deformed Cu fibers welded at the overlapping node during cooling process.

When using the die-casting process or the reactive metal penetration process, it is hard to guarantee the even distributing and continuous of metal phase. In addition, the fabricating efficiency of the die-casting process for composite substrates is limited [15,17]. Because fibers are easy to mix evenly with ceramic particles, the Al_2_O_3_/Cu ceramic composite with continuous Cu phase could be fabricated more simply and efficiently by the low-temperature hot-pressing process than by the die-casting or reactive metal penetration processes.

### 3.3. Thermal Performance

Ceramic-metal substrates with co-continuous ceramic-metal phases have both high thermal conductivity and compatible CTEs, but the die-casting infiltration process is too complicated to control composite quality and requires expensive equipment. The CTEs of Al_2_O_3_/30glass/Cu_f_ composites at 20 °C are shown in Table 3. With the increase in the Cu_f_ content, the CTE of the composites increased from 5.41 to 8.7 × 10^−6^/K, which is much lower than that of Cu (17.7 × 10^−6^/K) and matches well with that of the silicon chip (4.3 × 10^−6^/K).

**Figure 10:**
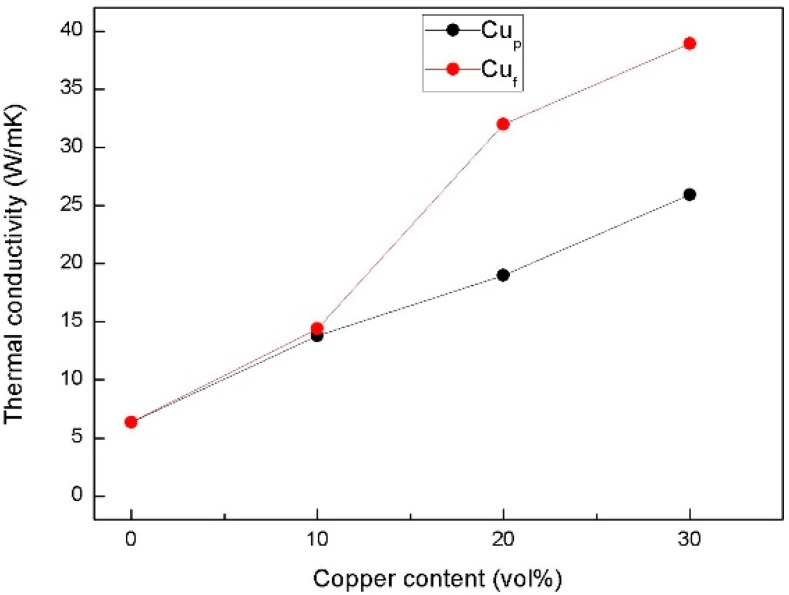
Thermal conductivity of Al_2_O_3_/30glass/Cu as a function of Cu content.

Figure 10 shows the thermal conductivity of Al_2_O_3_/30glass/Cu as a function of the Cu content at room temperature. When the contents of copper fibers or copper powders were less than 10 vol %, the thermal conductivity of Al_2_O_3_/30glass/Cu_f_ was similar to the thermal conductivity of Al_2_O_3_/30glass/Cu_p_, because at this content, the amount of copper fibers is too low to form the complete three-dimensional network structure in the composite. Similar to copper powders, copper fibers were distributed in a fragmented state in the composites. They were isolated by the glass and ceramic phases like Cu_p_ in composites. When the contents of Cu_f_ were more than 20 vol %, the enhancement of thermal conductivity by Cu_f_ was more remarkably improved than that by the same volume of Cu_p_, because the amount of copper fibers was large enough to ensure the copper fibers overlapping each other and to form a continuous 3D network structure in Al_2_O_3_/30glass/Cu_f_. When 30 vol % Cu was added, the thermal conductivity of Al_2_O_3_/30glass/Cu_f_ increased to 38.9 W/mK, while the thermal conductivity of Al_2_O_3_/30glass/30Cu_p_ was only 25.9 W/mK.

It is worthy of note that the thermal conductivity of the Al_2_O_3_/30glass/30Cu_f_ composite substrate by low-temperature sintering was also higher than that of Al_2_O_3_/30glass/30C_f_ (28.98 W/mK) [16], although the thermal conductivity of C fiber is higher than that of Cu fiber. The thermal conduction enhancement mechanism of Al_2_O_3_/30glass/30Cu_f_ composite substrate not only differs with that of Al_2_O_3_/30glass/30Cu_p_ composite substrate, but also differs from that of Al_2_O_3_/30glass/30C_f_ composite substrate. Similar to the carbon fiber 3D network in Al_2_O_3_/30glass/30C_f_ composite [16], Cu_f_ can form a bridging 3D network in the Al_2_O_3_/30glass/30Cu_f_ composite substrate, which would enhance the thermal conductivity effectively. When sintered at 850 °C under a pressure of 25 MPa, Cu_f_ was etched by the melted glass at local surface, leading to welding bridging in the overlapped fibers. The morphology of Cu phase changed from a fiber type to molten-like vermiform type. The molten vermiform-like Cu metallurgically bonded with each other (C_f_ connected physically) and formed a continuous network for rapid thermal conduction, which is very similar to the structure of ceramic-metal substrates prepared by the die-casting or reactive metal penetration process [15,17]. This is the main reason why the thermal conductivity of Al_2_O_3_/30glass/30Cu_f_ composite substrate is about 38.9 W/mK, but the thermal conductivity of Al_2_O_3_/30glass/30C_f_ is only 28.98 W/mK. Through the Cu fibers’ bridging thermal conduction mechanism, it was possible to manufacture a low-cost substrate with excellent thermal conductivity and moderate CTE for LEDs, integrated circuits and other industry fields.

Combining the microstructure observation, the thermal conduction mechanism of Al_2_O_3_/30glass/30Cu_f_ composite substrate could be deduced as follows:

(1) At the beginning of hot-pressing, copper fibers deformed and connected physically, forming a 3D network like that in the Al_2_O_3_/30glass/30C_f_ substrate. The oxide layer on the surface of copper fibers was destroyed by glass and Al_2_O_3_ particles.
(2) The surfaces of copper fibers tend to react with molten glass phase.
(3) Copper fibers achieved metallurgical bonding with each other and exhibited a molten-like vermiform shape.
(4) A continuous metallurgy 3D network of copper fiber was established, similar to that of die-casting or reactive metal penetration processes.

The novelty of this process lies in the fact that it could not only fabricate ceramic-metal substrate with excellent thermal properties, but also combines the advantages of the powder sintering process and the traditional die-casting penetration process for ceramic-based composite substrate with continuous metal phase. Cu_f_ in the composite could be mixed homogeneously, overcoming the thickness limitation of infiltration and the uneven distribution of the metal phase by the melt infiltration process. This fabricating process of Al_2_O_3_/30glass/30Cu_f_ provides a new way to mass-produce ceramic substrate with high thermal conductivity at low cost and high efficiency mode, which should be of much benefit to the industrial operations of the high-power LED and integrated circuits industry.

## 4. Conclusions

Al_2_O_3_/glass/Cu composite substrate with continuous metal phase was fabricated with copper fibers through a powder sintering process at low temperature. After hot-pressed sintering at 850 °C under a pressure of 25 MPa, the Cu_f_ morphology changed to a molten-like vermiform shape. The molten-like vermiform copper interconnected metallurgically and formed a continuous 3D network for rapid thermal conduction. Owing to the interconnected 3D network of the molten-like vermiform copper, the thermal conductivity of the Al_2_O_3_/30glass was enhanced from 6.35 W/mK to 38.9 W/mK with the addition of 30 vol % Cu_f_. The novelty of this process lies in the fact that it could not only fabricate ceramic-metal substrate with excellent thermal properties, but also combines the advantages of the powder sintering process and the traditional die-casting penetration process for ceramic-based composite substrate with continuous metal phase.

## Figures and Tables

**Table 1:** Composition of ZnO-SiO_2_-CaO glass (wt %).

ZnO	SiO_2_	CaCO_3_	Al_2_O_3_	TiO_2_	MgO	Na_2_CO_3_
20	45	18	6	2	8	1

**Table 2:** Composition of Al_2_O_3_/glass/Cu composites (in volume).

No.	Al_2_O_3_	Cu_f(p)_	Glass
F-0	70	0	30
F-1	60	10	30
F-2	50	20	30
F-3	40	30	30

**Table 3:** CTEs of the composite substrates.

Substrate	CTE (10^−6^/K)
F-0	5.41
F-1	7.6
F-2	8.01
F-3	8.7
